# Histological and parasitological distinctive findings in clinically-lesioned and normal-looking skin of dogs with different clinical stages of leishmaniosis

**DOI:** 10.1186/s13071-017-2051-6

**Published:** 2017-03-13

**Authors:** Laura Ordeix, Annabel Dalmau, Montsant Osso, Joan Llull, Sara Montserrat-Sangrà, Laia Solano-Gallego

**Affiliations:** 1 Departament de Medicina i Cirurgia Animals, Facultat de Veterinària, Universitat Autònoma de Barcelona, Bellaterra, Spain; 2 Hospital Clínic Veterinari, Universitat Autònoma de Barcelona, Bellaterra, Spain; 3 Mediterrani Veterinaris, Reus, Spain; 4 Consultori Veterinari, Falset del Priorat, Spain; 5 Hospital MonVeterinari, Manacor, Mallorca Spain

**Keywords:** *Leishmania infantum*, Dog, Inflammatory pattern, Skin, Quantitative PCR, Immunohistochemistry, Papular dermatitis

## Abstract

**Background:**

Normal-looking skin of dogs with leishmaniosis frequently shows microscopic lesions along with the presence of *Leishmania* amastigotes. However, histological lesions with or without detection of amastigotes might not occur in less severe clinical cases. In addition, comparative studies between paired clinically-lesioned and normal-looking skin samples from dogs with different disease severity are lacking. The objective of this study was to compare histological and parasitological findings by *Leishmania* immunohistochemistry (IHC) and quantitative PCR (qPCR) on paired clinically-lesioned and normal-looking skin biopsies from 25 dogs with different clinical stages of leishmaniosis, 11 with stage I-mild disease (papular dermatitis) and 14 with stage II-III (ulcerative or exfoliative dermatitis).

**Results:**

The study demonstrated microscopic lesions in 14 out of 25 (56%) samples from normal-looking skin biopsies. In those samples, perivascular to interstitial dermatitis composed by macrophages with lymphocytes and plasma cells was observed mainly in the superficial and mid-dermis. The intensity of the dermatitis was mild to moderate and always less prominent than in the clinically-lesioned skin. In normal-looking skin samples, the presence of parasites was detected by histology, IHC and qPCR in 5/25 (20%), 8/25 (32%) and 18/25 (72%), respectively. *Leishmania* was encountered in 11/25 (44%), 23/25 (92%) and 25/25 (100%) of clinically-lesioned skin samples by histology, IHC and qPCR, respectively. Normal-looking skin from dogs with stage I-mild disease was less frequently inflamed (*P* = 0.0172). Furthermore, *Leishmania* was more easily demonstrated by histology (*P* = 0.0464), IHC (*P* = 0.0421) or qPCR (*P* = 0.0068) in normal-looking skin of dogs with stage II-III-moderate to severe disease. In addition, in the latter group, there was a significantly higher parasite load studied by means of qPCR than in dogs with less severe disease (*P* = 0.043). Clinically-lesioned skin from dogs with stage I disease was more frequently characterised by the nodular to diffuse pattern and granuloma formation (*P* = 0.0166) and by a lower parasite load studied by means of qPCR (*P* = 0.043) compared with more diseased dogs.

**Conclusions:**

Normal-looking skin from dogs with stage I is less likely to present histological lesions as well as harbour the parasite when compared with dogs with moderate to severe leishmaniosis.

## Background

Canine leishmaniosis (CanL) caused by *Leishmania infantum* is a zoonotic vector-borne disease with a wide geographical distribution in both the Old and New World. Infected dogs are the main domestic reservoir of the parasite [1]. Dogs can manifest a chronic subclinical infection, self-limiting disease, or non-self-limiting illness [1, 2] as previously documented in humans [3]. Therefore, several degrees of disease severity are found in dogs ranging from mild disease to severe fatal disease. Two clinical staging systems are currently used in the clinical setting [2, 4]. LeishVet clinical staging system ranges from stage I-mild disease to stage IV-very severe disease with different clinical outcomes, prognosis and treatment options [2].

Cutaneous lesions are the most common clinical signs in CanL [5] and they are very pleomorphic from a clinical and histopathological point of view as well [6]. The most common dermatological signs observed in dogs with leishmaniosis include exfoliative dermatitis, ulcerative dermatitis and onychogryphosis [5]. However, other less typical manifestations such as papular dermatitis, muco-cutaneous nodular dermatitis or sterile pustular dermatitis are also diagnosed [5, 6]. This clinical variation is due to a wide variety of pathological mechanisms occurring secondarily to the inflammation, immune complex deposition and/or autoantibody production [7] and to the genetically determined or acquired inability of the immune system to control parasite multiplication and tissue invasion [8].

Among the cutaneous manifestations of CanL, papular dermatitis is the only permissible dermatologic manifestation in stage I leishmaniosis [2]. Dogs with papular dermatitis commonly show no other clinico-pathological abnormalities and anti-*Leishmania* antibodies are negative or weakly positive. This dermatological problem is associated with a good specific cell-mediated immune response as well as the spontaneous resolution of the lesions within 3–5 months in some cases [9–11].

The normal-looking skin has been scarcely studied either in diseased or in infected but clinically healthy dogs [12–15]. However, only one study evaluated both clinically-lesioned and normal-looking skin from the same individuals [14]. In addition, to the best of our knowledge, comparative studies between paired clinically-lesioned and normal-looking skin samples from dogs with different stages of disease severity are lacking. Normal-looking skin of dogs with leishmaniosis, with or without dermatological manifestations, frequently shows microscopic lesions along with the presence of *Leishmania* amastigotes [5]. However, this might not apply in less severe clinical cases.

The objective of this study was to characterise and compare the inflammatory pattern and the parasite burden by microscopic examination, immunohistochemistry (IHC) and real-time polymerase chain reaction (qPCR) analysis in paired clinically-lesioned and normal-looking skin from the same dogs with dermatological manifestations due to CanL with different stages of disease severity (stage I-mild disease *versus *stage II-III-moderate to severe disease).

## Methods

### Dogs and diagnosis of leishmaniosis

Twenty-five dogs with CanL and dermatological manifestation were prospectively enrolled at the time of diagnosis from January 2014 to February 2016. The dogs were from different Catalonian and Balearic veterinary centers from Spain: Fundació Hospital Clínic Veterinari (Bellaterra, Barcelona), Hospital Ars Veterinaria (Barcelona), Hospital Mediterrani Veterinaris (Reus, Tarragona), Consultori Montsant (Falset, Tarragona) and Hospital Mon Veterinari (Manacor, Mallorca)*.* The diagnosis of canine leishmaniosis was made based on the results of the physical examination and cytological or dermatopathological examination of cutaneous lesions. Moreover, a complete blood count using System Siemens Advia 120 (Siemens Healthcare GmbH, Germany), a biochemical profile including creatinine, urea, total proteins, alanine transaminase and total cholesterol by Analyzer Olympus AU 400 (Olympus, Center Valley, USA), serum protein electrophoresis by Hydrasys® (Sebia Electrophoresis, Lisses, France), urinalysis with urinary protein/creatinine ratio and quantitative serology for the detection of *L. infantum* specific antibodies by means of a serial dilution in-house ELISA were performed [16, 17]. Dogs were classified in four different stages (stage I-mild disease, II-moderate disease, III-severe disease and IV-very severe disease) at the time of diagnosis as previously described [2].

### Collection and processing of skin samples

Two skin fragments from paired clinically-lesioned and normal-looking skin were collected from each dog. Normal-looking skin was obtained whenever possible from the lateral aspect of the neck. In cases where this region was affected, normal-looking skin was collected as far away as possible from the macroscopic lesions. Each skin sample was then immediately cut into two halves. One half was fixed in 10% formalin for routine histological and immunohistochemical examination and the other one submerged in RNA later (RNAlater® Stabilization Solution, Ambion, Inc., Austin, Texas) and kept at -80 °C until used for RNA extraction and consecutively DNA purification for qPCR analysis.

### Histological examination and *Leishmania* immunohistochemistry

The dermal inflammatory pattern and the cell population were evaluated histologically in haematoxylin and eosin (HE)-stained sections. The distribution pattern of the infiltrate (perivascular to interstitial or nodular to diffuse with or without granuloma formation); the inflammatory cells (macrophages, lymphocytes, plasma cells and neutrophils); the degree (none, mild, moderate and severe) of cellular infiltration in the dermis and the epidermal changes (hyperplasia, spongiosis and exocytosis) were evaluated as previously described [18].

IHC for the detection of *L. infantum* amastigotes was performed as previously described [18]. The parasite load in immunolabelled sections was determined as the average number of microorganisms counted in five high power fields of areas with inflammatory infiltrate: 0, no microorganisms; 1, 1–10; 2, 11–30; and 3, > 30 [12].

### qPCR

RNA was isolated from skin biopsies using the RiboPure Kit (Ambion, Inc., Austin, Texas) and stored at −80 °C until used for future studies. DNA was purified from the interphase and organic phase generated from the RNA purification process by means of QIAamp DNA Mini Kit (Qiagen, Manchester, UK) following the manufacturer's instructions with slight modifications. Briefly, 20 μl of proteinase K solution and 200 μl of tissue sample were used in all cases. The other steps were performed as per manufacturer's protocol. A fragment of spleen and/or skin from a clinically healthy non-infected dog from a non-endemic area (United Kingdom) was used as a control for DNA contamination during DNA extraction.

qPCR was performed with a relative quantification as previously described with minor modifications [19]. Briefly, PCR mix reaction was prepared with 4 μl of DNA, 10 μl of master mix (TaqMan® Fast Advanced Master Mix, Thermo Fisher Scientific Inc.), 1 μl of *Leishmania* primers and probes (Custom TaqMan® Gene Expression Assay, ThermoFisher Scientific Inc., Waltham, USA) or 1 μl of another type of assay primers and probes [Eukaryotic 18S rRNA Endogenous Control (VIC™ ⁄ MGB Probe, Primer Limited, ThermoFisher Scientific Inc., Waltham, USA)] and 5 μl of H_2_O.

In order to verify that the PCR was done successfully, a positive control for *Leishmania* and a negative control from a non-infected clinically healthy dog were included in the plate. PCR was carried out in a QuantStudio Flex™ 7 Real-Time PCR system (ThermoFisher Scientific Inc., Waltham, USA). Thermal cycling profile consisted of 50 °C for 2 min in order to activate the enzyme called amperase and afterwards, a total of 40 cycles were carried out. Each cycle comprised 20 s at 95 °C followed by 40 cycles of 1 s at 95 °C and 20 s at 60 °C. To compensate for variations in total DNA input, mean values of cycle threshold (CT) from duplicate determinations from the *Leishmania* and 18S rRNA-PCR were taken for the calculation of the delta CT (difference of expression between *Leishmania* CT-18S rRNA CT).

### Statistical analysis

The statistical analysis was performed using the SPSS 22.0 for Windows software (SPSS Inc., USA). Categorical data were expressed as percentage and statistical analysis was performed using the McNemar's test and Fisher’s exact test to compare results among related or independent variables, respectively. Quantitative data were expressed as means and standard deviations and a non-parametric Wilcoxon signed-rank test and Mann-Whitney *U*-test were used to compare results among related or independent variables, respectively. Differences were considered significant with a 5% significance level (*P* < 0.05).

## Results

### Description of clinical data of dogs

Both sexes were represented by 11 females and 14 males. The median age was 2.5 years with a range from five months to 10 years. Eleven purebred dogs belonging to ten breeds and 14 mixed-breed dogs were included. Dogs were classified in three clinical stages: stage I-mild disease characterised by persistent papular dermatitis (11 dogs, six females and five males, median age 10 months), II-moderate disease (12 dogs, three females and nine males, median age 54 months) and III-severe disease (two female dogs, median age 54.5 months). For comparative analysis dogs were divided into two groups: group A (11 dogs with stage I) and group B (14 dogs with stage II and III). Age difference was statistically significant among groups (Mann-Whitney *U*-test, *Z* = -2.773, *P* = 0.006). In group A, six dogs were serologically negative, three were low positive and two medium positive, whereas in group B one was low positive, one was medium positive and 12 were high positive. Moreover, dogs from group A had significantly lower levels of *Leishmania* antibodies (136.8 ± 196.1 ELISA units, EU) than dogs from group B (8,892.7 ± 17,807.7 EU; Mann-Whitney *U*-test, *Z* = -3.747, *P* < 0.0001).

### Descriptive histopathology

#### Normal-looking skin

**Fig. 1:**
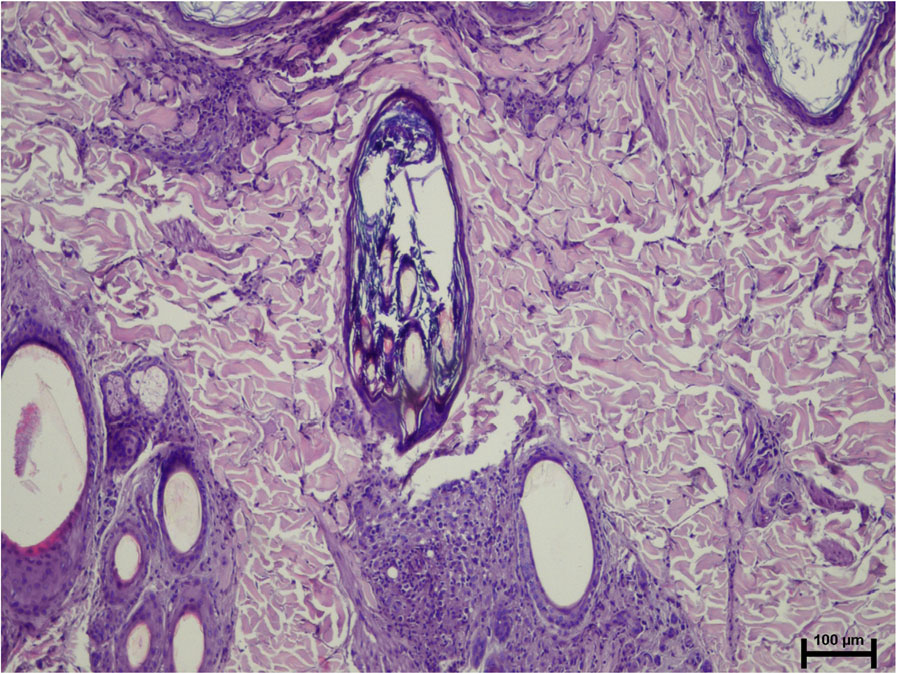
Superficial and mid perivascular to interstitial dermatitis in normal-looking skin from a dog with stage II leishmaniosis (haematoxylin and eosin staining)

**Fig. 2:**
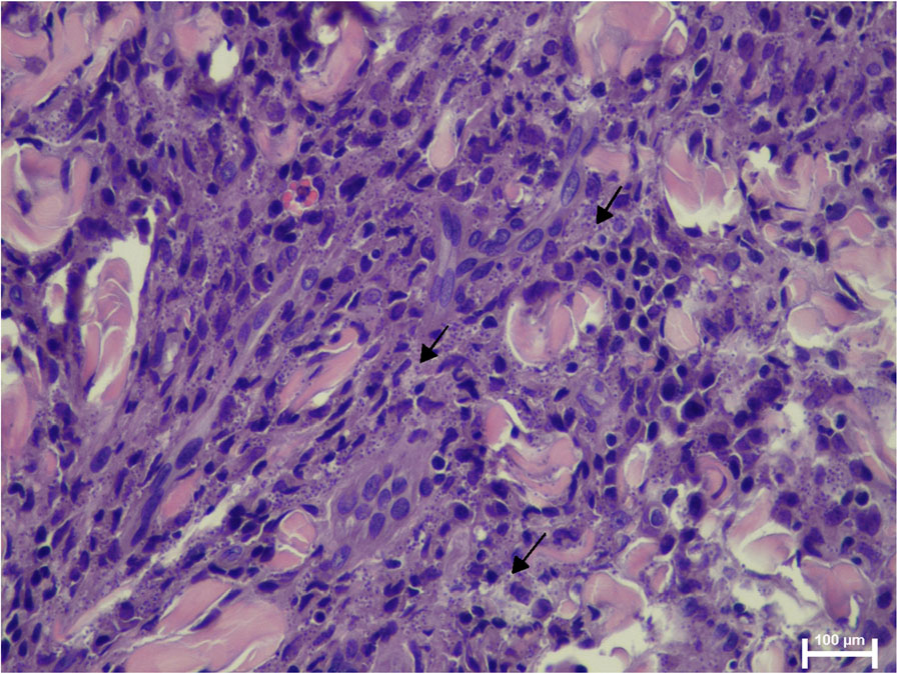
Numerous intracellular *Leishmania* amastigotes in macrophages (*arrows*) from the inflammatory infiltrate present in the dermis of normal-looking skin sample from a dog with stage II leishmaniosis (haematoxylin and eosin staining)

The prevalence of microscopic lesions and presence of *Leishmania* by means of HE in normal-looking skin samples are shown in Table 1. The epidermis was normal in all cases but one, with epidermal hyperplasia and ulceration. This case also showed moderate inflammatory infiltrate in the dermis with amastigotes visible with HE-stained sections. The inflammatory pattern observed ranged from perivascular to interstitial mainly in the superficial and mid-dermis in all cases (Fig. 1). The intensity of the dermatitis was mild to moderate in all cases where inflammation was present. Macrophages with lymphocytes and plasma cells were the predominant cells. In normal-looking skin samples, the detection of intramacrophagic structures compatible with amastigotes was demonstrated in 5/25 (20%) samples, all of them from dogs from group B (Fisher’s exact test, *P* = 0.0464) (Fig. 2).

**Table 1:** Frequency of microscopic lesions and detection of *Leishmania* by means of HE, IHC and qPCR on paired skin samples from the dogs studied based on disease stage. Values with the same superscript differ significantly

Skin samples	Microscopic lesions	Detection of *Leishmania*		
		HE	IHQ	qPCR
Normal-looking skin (*n* = 25)	14/25 (56.0 %)	5/25 (20%)	8/25 (32.0%)	18/25 (72.0%)
Stage I (*n* = 11)	3/11 (27.3%)^a,b^	0/11 (0%)^c^	1/11 (9.1%)^d^	5/11 (45.5%)^e^
Stage II-III (*n* = 14)	11/14 (78.6%)^b^	5/14 (35.7%)^c^	7/14 (50.0%)^d^	13/14 (92.9%)^e^
Clinically-lesioned skin (*n* = 25)	25/25 (100%)	11/25 (44.0%)	23/25 (92.0%)	25/25 (100%)
Stage I (*n* = 11)	11/11 (100%)^a^	1/11 (9.1%)^f^	9/11 (81.8%)	11/11 (100%)
Stage II-III (*n* = 14)	14/14 (100%)	10/14 (71.4%)^f^	14/14 (100%)	14/14 (100%)

*Abbreviations*: *HE* haematoxylin and eosin stained sections, *IHC Leishmania* immunohistochemistry, *qPCR* quantitative PCR

^a^McNemar's test: *P* = 0.008

^b^Fisher’s exact test: *P* = 0.0172

^c^Fisher’s exact test: *P* = 0.0464

^d^Fisher’s exact test: *P* = 0.0421

^e^Fisher’s exact test: *P* = 0.0068

^f^Fisher’s exact test: *P* = 0.0037

#### Clinically-lesioned skin

**Fig. 3:**
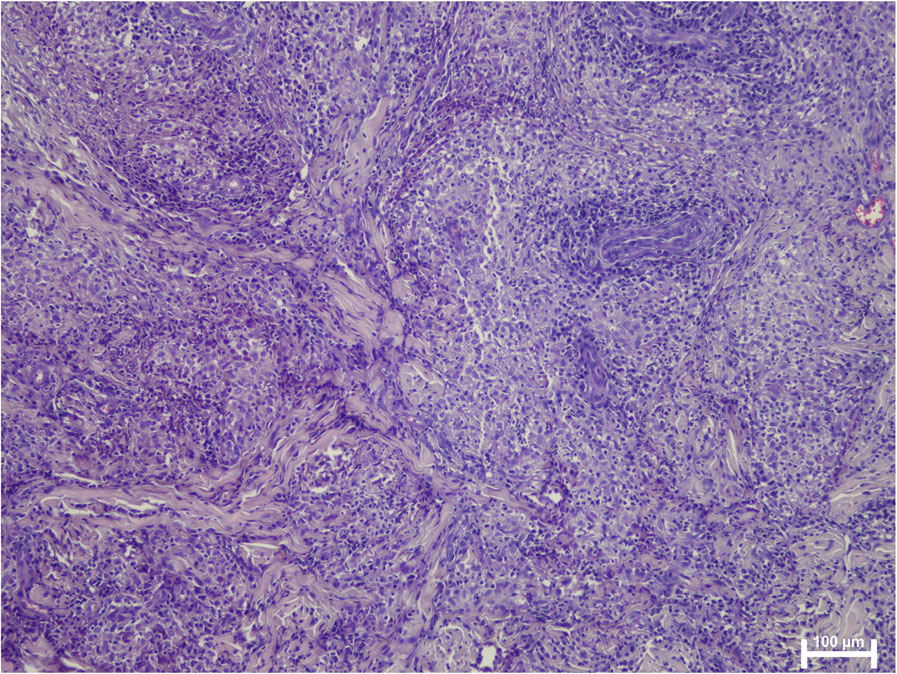
Nodular to diffuse dermatitis with granuloma formation in clinically-lesioned skin from a dog with stage I leishmaniosis (haematoxylin and eosin staining)

The prevalence of microscopic lesions and detection of *Leishmania* by means of HE in clinically-lesioned samples are shown in Table 1. The most common epidermal changes were hyperplasia (20/25), followed by ulceration (8/25) and hyperkeratosis (7/25). Only two samples had normal epidermis. Moderate to severe lympho-plasmacytic and macrophagic infiltrates were noted in the dermis of all patients together with few neutrophils in some patients. The inflammatory pattern observed was nodular to diffuse in 13 samples (nine from group A and four from group B) and perivascular to interstitial in 12 clinically-lesioned samples (two from group A and ten from group B). Therefore, skin samples from group A were more frequently characterised by a nodular to diffuse pattern than skin samples from group B (Fisher’s exact test, *P* = 0.0154). Granulomas were only observed in four samples, all of them from group A (Fisher’s exact test, *P* = 0.0166) (Fig. 3). Amastigotes compatible with *Leishmania* were noted in 11/25 (44%) samples. Most of these (10/11) were samples from group B and this difference was statistically significant (Fisher’s exact test, *P* = 0.0037).

#### *Leishmania* immunohistochemistry

**Fig. 4:**
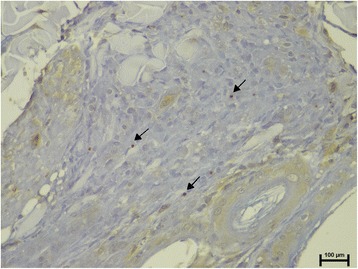
Few (1–10 per high power field) intracellular *Leishmania* amastigotes (arrows) are visualized in macrophages from the inflammatory infiltrate present in the dermis of normal-looking skin sample from the same dog as in Fig. 1 (*Leishmania*-specific IHC staining)

The prevalence of positive IHC in clinically-lesioned and normal-looking skin samples are shown in Table 1. Amastigotes were noted in 8/25 (32%) normal-looking skin samples. Seven out eight of these samples were from dogs from group B (Fisher’s exact test, *P* = 0.0421; Fig. 4). The majority of positive samples (6/8) had few amastigotes (1–10 per high power field) with one between 11–30 and another with more than 30 per high power field.

**Fig. 5:**
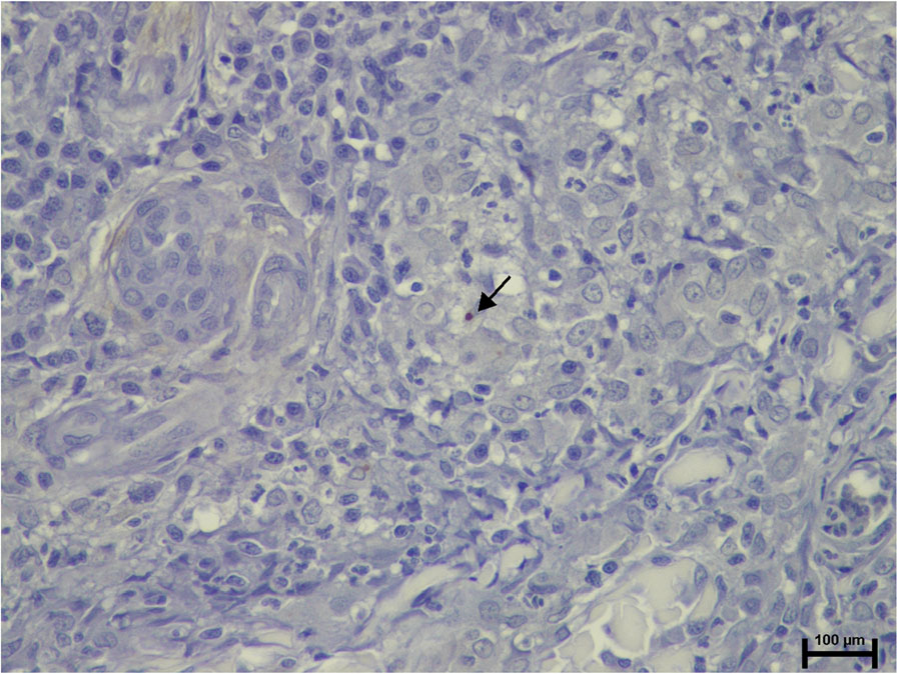
Note only one intracellular *Leishmania* amastigote (*arrow*) in the center of a granuloma in the inflammatory infiltrate present in the dermis of clinically-lesioned skin from the same dog as in Fig. 3 (*Leishmania*-specific IHC staining)

On the other hand, amastigotes were noted in 23/25 (92%) clinically-lesioned skin samples. Two samples with negative IHC were from dogs from group A. Although marginally statistically significant, there was a trend for a higher parasite load in clinically-lesioned skin from dogs from group B compared with group A (Mann-Whitney *U*-test: *Z* = -1,943, *P* = 0.052; Fig. 5; Table 2).

**Table 2:** Parasite load by means of *Leishmania-*specific IHC and qPCR on paired skin samples from the dogs studied based on disease stage

Skin samples	IHC^a^ (mean ± SD)	qPCR^g^ (mean ± SD)
Normal-looking skin (*n* = 25)	0.4 ± 0.8^b^	3.0 ± 4.7^h^
Stage I (*n* = 11)	0.1 ± 0.3^c,d^	6.1 ± 4.0^i,j^
Stage II-III (*n* = 14)	0.5 ± 0.7^d,e^	1.7 ± 4.5^j,k^
Clinically-lesioned skin (*n* = 25)	1.5 ± 0.9^b^	1.5 ± 4.9^h^
Stage I (*n* = 11)	1.1 ± 0.8^c,f^	3.4 ± 4.4^i,l^
Stage II-III (*n* = 14)	1.7 ± 0.9^e,f^	-0.4 ± 4.7^k,l^

*Abbreviations: qPCR*, quantitative PCR *IHC Leishmania* immunohistochemistry, *SD* standard deviation

^a^For method of grading, see Methods

^b^Wilcoxon Signed-rank test: *Z* = -4.345, *P* < 0.0001

^c^Wilcoxon Signed-rank test: *Z* = -2.887, *P* = 0.004

^d^Mann-Whitney *U*-test: *Z* = -2.169, *P* = 0.03

^e^Wilcoxon Signed-rank test: *Z* = -3.274, *P* = 0.001

^f^Mann-Whitney *U*-test: *Z* = -1.943, *P* = 0.052

^g^Delta CT (difference of expression between *Leishmania* CT -18S CT)

^h^Wilcoxon Signed-rank test: *Z* = -3.332, *P* = 0.001

^i^Wilcoxon Signed-rank test: *Z* = -2.023, *P* = 0.043

^j^Mann-Whitney *U*-test: *Z* = -2.021, *P* = 0.043

^k^Wilcoxon Signed-rank test: *Z* = -2.691, *P* = 0.007

^l^Mann-Whitney *U*-test: *Z* = -2.026, *P* = 0.043

#### qPCR

The normal-looking skin of 18/25 (72%) dogs studied was qPCR positive for *Leishmania* (Table 1). Negative qPCR was almost always associated with a microscopically normal skin. Only one dog presented mild perivascular dermatitis in the deep dermis and qPCR was negative. From 11 samples without histological lesions, five resulted qPCR positive. The prevalence of negative qPCR on normal-looking skin samples from dogs from group A was higher than that detected in normal-looking skin from dogs from group B (Fisher’s exact test, *P* = 0.0068). The parasite load studied by means of qPCR in normal-looking skin samples was always lower than in clinically-lesioned skin whatever the stage of disease (Wilcoxon signed-rank test, group A: *Z* = -2.023, *P* = 0.043; group B: *Z* = -2.691, *P* = 0.007; Table 2). The relative amounts of parasites in normal-looking skin from dogs from group A was lower than in normal-looking skin from dogs from group B (Mann-Whitney *U*-test: *Z* = -2.021, *P* = 0.043; Table 2).

As expected, 25/25 (100%) of clinically-lesioned skin were qPCR positive and the parasite load was higher in samples from dogs from group B compared with dogs from group A (Mann-Whitney *U*-test: *Z* = -2.026, *P* = 0.043, Table 2).

## Discussion

In this study, we demonstrated histological and parasite load differences not only among clinically-lesioned and normal-looking skin of the same dogs but also among skin samples of dogs with different clinical stages of leishmaniosis.

In agreement with previous studies, we demonstrated that the normal-looking skin of dogs with leishmaniosis frequently shows microscopic lesions (56%) and harbours the parasite, as demonstrated by routine HE staining (20%), *Leishmania*-specific IHC (32%) and, more often, by qPCR (72%). However, there are some differences among our results and those previously reported [12–15]. The prevalence of microscopic lesions and detection of amastigotes either by routine histology or by IHC in our study was at the lower limit of the ranges reported in previous studies. Microscopic lesions have been noticed in 50–100% of the skin samples obtained from the normal-looking skin of dogs with CanL [5, 12–14]. Moreover, amastigotes were seen in up to 100% of the cases, depending on the sensitivity of the method employed [5]. These findings are probably related to the fact that in the present study about half of the dogs had mild disease, i.e. papular dermatitis. Conversely, previous studies included either dogs with more severe disease, i.e. exfoliative dermatitis [14] or even stray dogs, which could present co-factors, such as co-infections or malnutrition, affecting the severity of disease [12, 13].

In the present study, we demonstrated that dogs with different clinical stages of leishmaniosis presented differences in the frequency of microscopic lesions and parasite load in normal-looking skin. The skin biopsies from normal-looking skin from dogs with stage I-mild disease (papular dermatitis) were significantly less frequently inflamed. Furthermore, *Leishmania* was more frequently demonstrated by routine histology, immunohistochemical examination or qPCR in normal-looking skin of dogs with stage II-III-moderate to severe disease. In addition, in the latter group, there was a significantly higher parasite load studied by means of qPCR than in dogs with less severe disease. These results suggest that dermal inflammation and cutaneous parasitism in normal-looking skin were directly related to the severity of clinical disease. Normal-looking skin of dogs with stage I-mild disease may resemble the skin of seronegative infected but clinically healthy dogs that is characterized by no histological lesions and absence of parasites by IHC, although their presence can be demonstrated by PCR [12].

Microscopic lesions and presence of amastigotes in the inflammatory infiltrate in normal-looking skin of diseased dogs is suggestive of haematogenous dissemination of the parasite and tropism for the skin [12]. Moreover, it has been demonstrated that dissemination to the skin varies between dogs, being greater in sick and infectious dogs [20]. Therefore, lack of these changes in the majority of dogs with normal-looking skin with stage I-mild disease would further suggest a protective immune response in these dogs able to control parasite dissemination at the site of parasite inoculation and multiplication as previously proposed [11, 18].

Histological findings observed in clinically-lesioned skin of dogs included in this study were in accordance with the literature [5, 6, 18] and amastigotes were variably seen in 44 and 92% of the cases, depending on the method employed. However, the results of this study further confirm that skin biopsies from dogs with papular dermatitis (stage I-mild disease) are characterised by the nodular to diffuse pattern and a significant higher frequency of granuloma formation compared with more severe cutaneous manifestation of CanL (stage II–III- moderate or severe disease) [18]. It has been proposed previously that there is a trend for a lower parasite burden in skin samples from dogs with stage I-mild disease [18]. Although amastigotes were more frequently noted in HE stained slides from stage II-III diseased dogs when compared with stage I dogs, there were no statistically significant differences in prevalence between positive IHC or qPCR among both groups studied. Nevertheless, the parasite load studied by means of qPCR was lower in samples from dogs with stage I-mild disease compared with dogs with severe disease. Taken together, these data might reinforce the idea of a protective immune response that these dogs have as described elsewhere [10, 11, 18].

Several studies have focused on the capacity of dogs to infect phlebotomine sand flies. It has been reported that the proportion of infected sand flies increases with the appearance and severity of the clinical signs and that good predictors of infectiousness are antibody levels and clinical disease, since no dogs have been found to be infectious before the detection of anti-*Leishmania* IgG antibodies [21, 22]. Moreover, it has been recently suggested that high parasite loads in dog ear skin, rather than the simple presence of parasites, is the most important metric to identify likely infectious individuals and potential reservoir populations [20]. Therefore, the fact that dogs with stage I-mild disease or papular dermatitis are characterised by reduced parasite load in both normal-looking skin and clinically-lesioned skin, emphasizes the concept that these dogs do not play a significant role in *L. infantum* infection of phlebotomine sand flies as opposed to dogs with stage II-III disease.

## Conclusions

In conclusion, this study confirms that normal-looking skin from dogs with stage I is less likely to present microscopic lesions as well as harbour the parasite when compared with dogs with moderate to severe CanL. Moreover, clinically-lesioned skin from dogs with stage I shows a lower parasite load than clinically-lesioned skin from more diseased dogs.

## Abbreviations

CanL: Canine leishmaniosis
CT: Cycle threshold
ELISA: Enzyme-linked immunosorbent assay
IHC: Immunohistochemistry
QPCR: Quantitative polymerase chain reaction
